# Omega-3 Versus Omega-6 Polyunsaturated Fatty Acids in the Prevention and Treatment of Inflammatory Skin Diseases

**DOI:** 10.3390/ijms21030741

**Published:** 2020-01-23

**Authors:** Anamaria Balić, Domagoj Vlašić, Kristina Žužul, Branka Marinović, Zrinka Bukvić Mokos

**Affiliations:** 1 Department of Dermatology and Venereology, University Hospital Centre Zagreb, School of Medicine University of Zagreb, Šalata 4, 10 000 Zagreb, Croatia; jovicanamaria@gmail.com (A.B.); branka.marinovic@kbc-zagreb.hr (B.M.); 2 Department of Ophtalmology and Optometry, General Hospital Dubrovnik, Ulica dr. Roka Mišetića 2, 20000 Dubrovnik, Croatia; domagojvlasic@yahoo.com; 3 School of Medicine, University of Zagreb, Šalata 3, 10000 Zagreb, Croatia; zuzulkristina@gmail.com

**Keywords:** nutraceuticals, polyunsaturated fatty acids, supplementation, eicosapentaenoic acid, docosahexaenoic acid, gamma-linolenic acid, inflammatory skin diseases, atopic dermatitis, acne, psoriasis

## Abstract

Omega-3 (ω-3) and omega-6 (ω-6) polyunsaturated fatty acids (PUFAs) are nowadays desirable components of oils with special dietary and functional properties. Their therapeutic and health-promoting effects have already been established in various chronic inflammatory and autoimmune diseases through various mechanisms, including modifications in cell membrane lipid composition, gene expression, cellular metabolism, and signal transduction. The application of ω-3 and ω-6 PUFAs in most common skin diseases has been examined in numerous studies, but their results and conclusions were mostly opposing and inconclusive. It seems that combined ω-6, gamma-linolenic acid (GLA), and ω-3 long-chain PUFAs supplementation exhibits the highest potential in diminishing inflammatory processes, which could be beneficial for the management of inflammatory skin diseases, such as atopic dermatitis, psoriasis, and acne. Due to significant population and individually-based genetic variations that impact PUFAs metabolism and associated metabolites, gene expression, and subsequent inflammatory responses, at this point, we could not recommend strict dietary and supplementation strategies for disease prevention and treatment that will be appropriate for all. Well-balanced nutrition and additional anti-inflammatory PUFA-based supplementation should be encouraged in a targeted manner for individuals in need to provide better management of skin diseases but, most importantly, to maintain and improve overall skin health.

## 1. Introduction

Since 1929, when Burr and Burr first described a syndrome caused by stringent fat reduction in a diet (which manifested mostly as cutaneous symptoms, such as erythema with scaling, hair loss, itch, and increased water loss), it became clear that particular fat pay an essential role in skin structure [1,2]. The word “essential” best describes these fats because of the inability of the human organism to synthesize them, which means they can only be provided through dietary intake. The term essential fatty acids (EFAs) was formed and referred to two polyunsaturated fatty acids (PUFAs), linoleic acid (LA) and α-linolenic acid (ALA), initiating acids for the cascade of elongation to very long-chain PUFAs (more than 22 C-atoms). PUFAs are divided into two families, omega-3 (ω-3) and omega-6 (ω-6). ω-3 fatty acids (FAs) have in common a terminal carbon-carbon double bond in the omega three-position, the third bond from the methyl end of the acid, whereas, ω-6 acids have it in the omega six-position, the sixth bond from the methyl end of the fatty acid, respectively. LA is a member of the ω-6 family, whereas, ALA is classified as ω-3 PUFA. The double bonds in these EFAs are always in *cis*-configuration, which means there are two hydrogen atoms on the same side of the double bond [3]. Salubrious effects of PUFAs might be mediated through various mechanisms, including modifications in cell membrane lipid composition, gene expression, cellular metabolism, and signal transduction [4]. However, ω-3 and ω-6 FAs have antagonistic effects on metabolic functions in the human organism.

PUFAs are nowadays desirable components of “specialty oils”, oils with special dietary, and functional properties that are used as nutraceuticals or cosmeceuticals. Due to the better understanding of their biological and functional properties, and their health benefits, PUFAs, specially ω-3 PUFAs are of great importance for health system, due to their potential applications in disease prevention, but also treatment of the most common chronic inflammatory diseases, including inflammatory skin diseases, such as atopic dermatitis (AD), psoriasis, and acne. Nowadays, clinicians have at the disposal useful tools like lipidomics and nutrilipomics that guide them to provide the most appropriate and individualized FAs supplementation in the treatment of their patients, but also the prevention of disease in various clinical fields, as well as the field of dermatology [5].

## 2. Sources of PUFAs

High dietary intake of ω-6 is typical to the Western diet, which is loaded with processed food and lacks fish meals as opposed to high red meat dietary intake [6]. Most seed and vegetable oils (safflower, grape seed, rapeseed, poppy seed, sunflower, palm, hemp, corn, wheat germ, cottonseed, soybean) which are used in the kitchen are significant sources of ω-6 PUFAs in the form of LA with low proportions of ω-3 FAs, first to mention—ALA. In contrast to ω-6 FAs, the intake of ω-3 is usually insufficient because of limited sources. ALA is found in green leafy vegetables, flaxseed, walnuts, soybean, and canola oils. Their derivatives, eicosapentaenoic acid (EPA) and docosahexaenoic acid (DHA), are obtained through breast milk and the fish oils, such as salmon, mackerel, sardines, anchovies, herring, and rainbow trout, but also algae [7,8,9,10]. Cold-water marine (wild) fishes are abundant in ω-3 PUFAs, since most of them feed on phytoplankton and zooplankton, a rich ω-3 PUFA source. ω-6 PUFAs, gamma-linolenic acid (GLA) that is typically consumed as part of dietary supplementation, is found in human milk and some botanical seed oils as borage oil, blackcurrant and evening primrose oil (EPO), while arachidonic acid (ARA) is obtained from diet rich in organ meats, poultry, and eggs. In typical Western diets, the ω-6/ω-3 ratio is 15/1 to 16.7/1, although the recommended ratio varies from 1:1 up to 4:1, as recommended by authoritative bodies in Japan (Table 1). In plants and animal-based food majority of PUFAs are found in the form of triacylglycerols (TAG), phospholipids (PL), diacylglycerols (DAG), and cholesterol esters (CE). PLs are the most bioavailable because of their aliphatic characteristics, which lead to better water dispersibility and greater susceptibility [11]. Therefore, krill oils obtained from Antarctic krill (*Euphausia superba*), which are fulfilled of ω-3 PUFAs in PL form, are becoming more popular as a ω-3 supplement. In addition to food supplements, we can now find many new conventional foods and infant feeding formulas fortified with microalgae and fish DHA [12].

## 3. Metabolism of PUFAs

**Figure 1:**
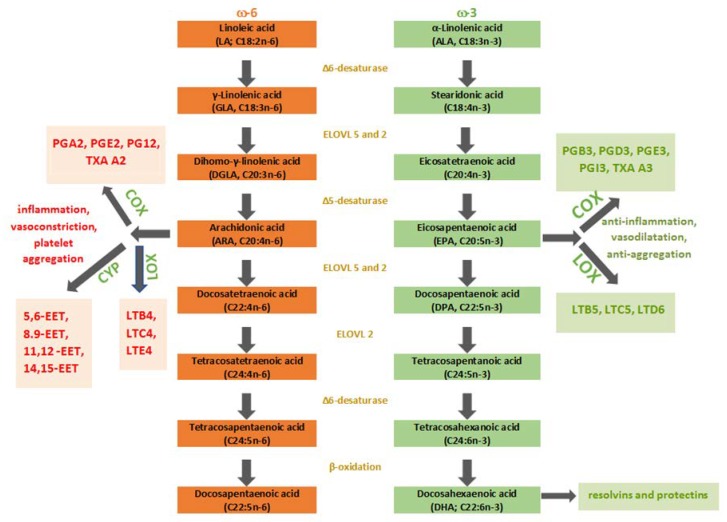
Schematic presentation of the PUFAs pathway. ω-3, omega-3 fatty acids; ω-6, omega-6 fatty acids; COX, cyclooxygenase; CYP, cytochrome P450; EET, epoxyeicosatrienoic acid; ELOVL, elongase; LOX, lipoxygenase; LT, leukotriene; PG, prostaglandin; TXA, thromboxane.

The ω-6 and ω-3 FAs, when consumed in food, are mostly assimilated into TAG and undergo digestion in the small intestine. The destruction of TAG structure and liberation of FAs allows absorption and transport in the bloodstream to the tissues where they are incorporated in their structure. The FAs can take three different metabolic pathways: (a) esterification into cellular lipids as PL, TAG, CE; (b) beta-oxidation to provide energy for ATP formation; (c) being an initiating structure for the process of elongation and desaturation through enzymatic reactions to create long-chain PUFAs. The distribution of FAs into PL is nowadays considered to be the most important factor that drives the cell membrane composition and consequent cell membrane homeostasis maintenance obtained by the membrane balance of FAs, but also FAs detachment of the PL for becoming signaling mediators. Nutrition, metabolism, external and internal stressors with consequent oxidative stress, and lifestyle factors all cause FAs changes that result in the impaired cell membrane homeostasis. This is why FAs are nowadays considered as one of the biomarkers of pathological conditions and why it is important to address the role of fatty acid-based membrane lipidomics, a powerful diagnostic tool for determining quantitative and qualitative changes of FAs, but also the follow-up of the membrane FAs remodeling associated with different physiological and pathological conditions [15]. The creation of the long-chain PUFAs occurs mostly in the liver. Other tissues are active in this process in an insignificant amount [3]. The most prominent and metabolically significant PUFAs derived from LA are GLA, dihomo-gamma-linolenic acid (DGLA), and ARA. On the other hand, the ω-3 family is derived from ALA, and the most notable among them are EPA and DHA. In the enzymatic cascades, eicosanoids are produced from the latter PUFAs (Figure 1). Eicosanoids are defined as bioactive signaling molecules with pro-inflammatory and anti-inflammatory characteristics, which are derived from ARA, DGLA, EPA, and DHA through reactions led by cyclooxygenase (COX-1 and COX-2), lipoxygenase (5-LOX and 15-LOX) and epoxygenases (cytochrome P450 or CYP). LA and ALA compete in the synthesis of eicosanoids by targeting the same enzymes, precisely desaturase and elongase enzymes, to become C20 FAs, which are the precursors of eicosanoids—oxygenated derivates of C20 FAs made by oxygenase enzymes. Higher intake of ALA results in the increased production of anti-inflammatory eicosanoids because ω-3 FAs are more favored substrates for desaturase and elongase enzymes than ω-6 FAs in eicosanoid synthesis. LA can be converted to ALA in the majority of higher plants, algae, and fungi because they possess Δ12- and Δ15-desaturase [16]. Nevertheless, in mammals, these enzymes are absent for genetic reasons, which makes humans unable to convert oleic acid (OA) to ALA and LA. This enzyme deficit makes the latter PUFAs essential. The level of EPA and DPA in the organism is mostly determined by direct dietary consumption because most of dietary ALA undergo beta-oxidation in the mitochondria [3]. Desaturation led by Δ6-fatty acid desaturase (FADS) makes the first step in long-chain PUFA synthesis by adding a double bond at the 6th C-C position from the -COOH of LA and ALA generating GLA and stearidonic acid (SDA), respectively. Δ6-FADS is a crucial enzyme in the long-chain (LC)-PUFAs synthesis because it is rate-limiting in animals and humans. The next step is elongation, and DGLA and eicosatetraenoic acid (ETA), respectively, are synthesized. Finally, Δ5-FADS adds a new double bond to the 5th C-C bond from carboxy-end, which results in the generation of ARA and EPA, respectively. Desaturation on the 17th C-C position results in GLA, DGLA, and ARA conversion to SDA, ETA, and EPA, respectively. FADS, crucial enzymes of rate-limiting steps in the LC-PUFAs biosynthesis pathway, are encoded by fatty acid desaturase 1 (*FADS1*) and 2 (*FADS2*) genes located on chromosome 11 (*FADS* cluster). A mammalian organism can synthesize DHA through three steps (two elongations and beta-oxidation) from EPA. This synthesis is also known as the Sprecher pathway [17]. The EPA and DHA formation take place in human hepatoma cells at the highest rate when the ratio 1:1 = ALA: LA is present [18]. The recorded conversion rates were 16% for EPA and 0.7% for DHA, which leads to the conclusion that DHA supplementation is the most effective way to improve body DHA levels. Biosynthesised PUFAs (ARA, DGLA, EPA, DHA) are stored in esterified form in PL or as neutral glycerides and can be mobilized when needed by phospholipase A2 as free (unesterified) FAs to form eicosanoids or other autacoids by oxygenase enzymes [19]. COX-2 forms series two prostaglandins (PG) from ARA, whereas, lipoxygenase (5-LOX), forms series four leukotrienes (LT) (B4, C4, E4). On the other hand, EPA is metabolized to series three (B3, D3, E3, I3) PG and series five LT (B5, C5, and D6) with their potent anti-inflammatory, vasodilatory, and anti-aggregative functions [20]. Protectins, D-series resolvins, and maresins are autacoids, which are the product of DHA metabolism. To some extent, we can say that ω−6 PUFAs derived eicosanoids are pro-inflammatory, whereas, ω−3 PUFAs derived eicosanoids have an anti-inflammatory role; the ratio ω−6/ω−3 PUFAs in a diet mainly induces the production of pro-inflammatory and anti-inflammatory eicosanoids which regulate homeostatic and inflammatory processes connected with infection, inflammation and cancer formation [19,21]. Although ω−6 FAs and their derivates are in general considered as ‘‘bad’’ omegas mainly because of the ARA and its products that enhance inflammation in numerous cell types and disease states, ARA’s substrate DGLA (ARA is synthesized from DGLA via Δ5-FADS) has long been considered as potent anti-inflammatory PUFA due to the oxygenated derivates—series-1 PGs, particularly PGE1 and 15-hydroxyeicosatrienoic acid (15-HETrE) that both antagonize the synthesis of ARA-derived pro-inflammatory eicosanoids [22,23].

## 4. PUFAs as Gene Expression Regulators

PUFAs are found to be significant gene modulators that regulate the expression of proteins related to inflammation and lipid metabolism [3]. Depending on the specific cell/tissue context and target gene, PUFAs and their oxidized metabolites might use different routes to regulate transcription and consequent cellular activities via nuclear and cellular receptors [22,24]. According to Deckelbaum et al. [25], the PUFAs affect sterol regulatory element-binding protein (SREBP)-depended gene expression. Namely, by activating the cellular cascade in state of sterol deprivation, a transcriptionally active amino-terminal fragment of SREBP (n-SREBP) is released and binds to SRE in the promoter region of many genes of lipid metabolism. The cascade begins in the endoplasmatic reticulum (ER). Cholesterol and oxysterols are critical regulators of this process as they act as end-product feedback inhibitors [26]. EPA, DHA, and ARA affect this process by decreasing the affinity of cholesterol for PL, resulting in enhancing its transfer from cholesterol-abundant regions (for example, cell membrane) to cholesterol-lacking regions (such as ER) [27]. This indirect inhibition orchestrated by PUFAs results in decreased SREBP transport out of ER to Golgi and, consequently, the absence of n-SREBP release. The other way how PUFAs can regulate gene expression includes activation of transcription factors via peroxisome proliferator-activated receptors (PPARs). PPARs are present as three types: PPAR-α, PPAR-β/δ, and PPAR-γ with its three isoforms: γ1, γ2, γ3. They are the members of the nuclear receptor family with tissue-specific expression and ligand-specific activation which pairs with the retinoic acid X receptor (RXR) and bind to specific regions on the DNA of target genes to achieve their comprehensive actions—increasing transcription of specific genes and decreasing transcription of others involved in the regulation of cellular differentiation, development, carbohydrate, lipid and protein metabolism, and tumorigenesis. The function of PPARs is orchestrated by the precise shape of their ligand-binding domain induced by ligand binding and by a vast number of proteins, coactivators, and corepressors, responsible for the stimulation of receptor function, respectively [28]. Although they have been used in endocrinology as lipid and insulin regulators for a long time, their use in dermatology is still modest. This diffident use in treating skin diseases is about to change because it has been discovered, regarding the skin tissue, that keratinocytes, Langerhans cells (LHCs) and melanocytes express all three PPAR isoforms [29,30,31] The most interesting among them in terms of use in dermatology is PPAR-γ, due to its potent anti-inflammatory properties. The best-known PPAR-γ agonists are thiazolidinediones, a group of drugs known as glitazones that are being used in endocrinology for decades. Also, many naturally occurring agents directly bind with and activate PPAR-γ; PUFAs and their metabolites, eicosanoids, are one of them. Binding of PPAR-γ to its coactivators also appears to reduce the levels of these coactivators available for binding to pro-inflammatory transcription factors, such as nuclear factor kappa B (NF-κB) which results in PPAR-γ transpression, repression of key inflammatory transcription factors of various pro-inflammatory genes, including tumor necrosis factors (TNF) and interleukins (IL). Activation of PPAR-γ inhibits TNF-alpha (TNF-α) formation from adipocytes [32]. It has also been found that in skin PPAR-γ has an inhibiting effect on macrophage-induced interferon-gamma (IFN-γ) production [33,34] and secretion of IL-12 from dendritic cells (DCs) [35,36]. The researches also proved PPAR-γ as an inhibitor of the LOX activity [37]. The latter mentioned anti-inflammatory properties of PPAR-γ make it an exciting candidate for psoriasis, AD, acne, and suppurative hidradenitis treatment. ω-3 PUFAs, EPA and DHA, being natural PPAR ligands, serve as natural mediators between PPAR signaling, SREBP expression, and liver-X-receptor [38,39,40]. These two ω-3 PUFAs are natural PPAR ligands. Their ability to inhibit inflammation and suppress genes related to lipid metabolism makes them an excellent and valuable agent in dyslipidemia, type 2-diabetes, cardiovascular, and inflammatory skin diseases. This mechanism has been proved when EPA and DHA manifested similar regulatory effects as glitazone on numerous genes and transcription factors [41,42]. 

## 5. Toll-Like Receptors and PUFAs—Modulating Immune Response

The recent findings have shown the importance of immune response modulation by activating/inhibiting Toll-like receptors (TLRs), a group of membrane-spacing protein receptors referred to as pattern-recognition receptors (PRRs), involved in the first-line recognition of pathogens that are also considered as critical proteins linking innate and acquired immunity [43,44]. The TLRs are present on a vast number of cells in the human body, including monocytes, macrophages, DCs, LHCs, natural killer cells (NKCs), granulocytes, and non-immune cells as endothelial and epithelial cells, as well as fibroblasts and adipocytes. Regarding the skin, keratinocytes, monocytes, sebocytes, LHCs, and dermal DCs all exhibit TLRs [45]. There are minimal 10 TLRs present in humans [45,46]. They are divided into three categories: lipopeptide-sensing TLRs (1, 2, 4, and 6), protein-sensing TLRs (5), and nucleic acid-sensing TLRs (3, 7, 8, and 9) [46]. The most important epidermal cells expressing TLRs include keratinocytes, which express TLRs receptors 1-6 and 9, and LCs that can express all TLRs, particularly TLRs 1, 2, 3, 5, 6, and 10 [47]. TLRs can distinct signals from recognized commensal organisms, where tolerance has been formed upon them through evolution, or when danger is sensed, start inflammatory response leading to DCs maturation and adaptive immuno-response activation. The intracellular transmembrane component of TLR is homologous to the IL-1 receptor, which leads to the activation of the NF-κB pathway [48]. Besides the best-known NF-κB pathway, in TLRs signaling, there is the activation of some other kinases, such as extracellular signal-regulated kinase (ERK) 1 and 2, p38 mitogen-activated protein kinases (MAPKs) and c-Jun-NH2-terminal kinases. By activating the TLRs pathway, pro-inflammatory cytokines and antimicrobial peptides are produced. As we mentioned before, besides their critical role in innate immunity, TLRs control, to some extent, B and T cell adaptive immune responses, as well as humoral ones. TLRs exhibit their actions related to the adaptive immune responses through the promotion of upregulation of CD80, CD40, and CD86, and the production of interleukin IL-12 by DCs and monocytes which participates in the differentiation of naive T cells into T helper 1 (Th-1) cells, inducing Th-1 cell-mediated immune responses. DHA and EPA, as natural TLRs ligands, can reduce the pro-inflammatory signals from TLR-2 in human monocytes [41]. Furthermore, both DHA and EPA inhibit TLR-4, the receptor for lipopolysaccharide (LPS), both exhibiting inhibitory effect on LPS-induced NF-κB activation and subsequent production of inflammatory cytokines (TNF-α, IL-1, IL-6, IL-8, and IL-12), CPX-2, and inducible nitric oxide synthase (iNOS) in various cells, due to decreased MAPKs activation via G-protein coupled receptor (GPR) 120 which results in decreased IκB phosphorylation [46,49]. Results of Mobraten et al., who examined the LC-PUFAs in the treatment of inflammatory bowel disease (IBD), showed that both ω-3 (EPA, DHA) and ω-6 PUFAs (ARA), natural GPR120 ligands, induce the same GPR120-mediated anti-inflammatory signaling events, but with different kinetics and efficiency in intestinal epithelial Caco-2 cells [50]. Since TLRs expression has been demonstrated in the skin where host-pathogen interactions are of great importance, they play an important role in the pathophysiology of the most common inflammatory skin diseases likeAD, psoriasis, acne, as well as skin infections [51,52]. At the same time, this is an important field where beneficial properties of PUFAs could be implemented. 

## 6. Skin and PUFAs

The production of LC-PUFAs derivatives is minimal in the skin, due to the absence of Δ5- and Δ6-FADS enzymes. These enzymes are crucial in synthesis cascade, which results in the biosynthesis of inflammatory modulators, eicosanoids, and autacoids. Besides being insufficient, they are sensitive to external influences. Namely, trans-fats, such as fat-free and glucose-rich diets, alcohol, corticosteroids, hypoinsulinemia, hypothyroidism, age, smoking, vitamin B6 and zinc deficiency, reduce the activity of the desaturase enzymes. These facts lead to a low conversion rate of ALA and LA [18]. The epidermis is composed of cells and lipid-rich extracellular matrix (ECM), with LA being the most prominent FA. It is a precursor to ceramides, a significant construction component of the matrix, which forms a stratum corneum permeability barrier (SCPB). The SCBP consists of three principal components: The extracellular lipid matrix, the cornified envelop, and thick keratin fibrils aggregated by filaggrin protein [53]. The extracellular lipid matrix consists of 50% ceramides, 25% cholesterol, and 15% free FAs [54]. In between lamellar bodies in upper layers of the epidermis [stratum spinosum and granulosum] lipids, enzymes, and antimicrobial peptides can be found [53]. The robust watertight lamella is formed by an extracellular lipid matrix overlaying the cornified envelop proteins. SCBP constructional defects are a result of mutations of the genes which code proteins and enzymes or in lipid deprivation state [54]. The epidermis can synthesize monounsaturated and saturated FAs, in contrast to PUFAs, which have to be acquired from the diet, due to the absence of Δ6- and Δ5-elongase [55]. The experiments discovered proteins that are responsible for the transport of dietary FAs and cholesterol to the stratum corneum. These FA-transport proteins are present in keratinocytes [56,57,58,59,60,61]. Furthermore, it has been proved that they are more selective for PUFAs than monounsaturated FAs. 

## 7. Atopic Dermatitis and PUFAs

AD is the most common chronic inflammatory skin disease with increasing prevalence, characterized by strong genetic background, chronic or chronically relapsing course, pruritus, and typical eczematous morphology with age-specific distribution patterns [62]. The pathophysiology of AD is complex and multifactorial, but in general, can be divided into three major categories which are each modulated by genetic and environmental factors—epidermal barrier dysfunction, immune dysregulation, and alteration of the microbiome [63]. Loss of function mutations in filaggrin has been implicated in severe aAD, due to a potential increase in trans-epidermal water loss (TEWL), pH alterations, and dehydration. Other genetic changes have also been identified, which may alter the skin’s barrier function, resulting in an ADphenotype. [64]. Filaggrin is considered essential for the aggregation of keratin filaments in the stratum corneum. The adequate ceramide production and consequently rigid SCPB construction are depended upon the delivery of acid derivatives. Besides physiological filaggrin structure, an effective permeability barrier is the result of synchronized enzyme reactions. Although a defect barrier in AD makes an ideal entry point for continual antigen invasion and inflammation, patients with inherited filaggrin defects in ichthyosis vulgaris do not experience high inflammation levels, showing the importance of immunity in AD, both innate and adaptive immune systems and their dynamic, interrelated roles [65]. We can say that AD is an inflammatory disease spectrum driven not by a single polar immune pathway, but multiple immune pathways responsible for different clinical features. There is a predominance of the Th2 inflammatory pathway in acute AD lesions characterized by elevated IL-4, IL-5, IL-13, IL-31, subsequent activation of mastocytes and eosinophils, and the production of allergen-specific IgE. Parallel to the chronicity of lesions, Th2 cytokine profile changes to Th1 and Th 22 profile, but also variable levels of Th17 cytokines in both acute and chronic inflammation that are important in the regulation of innate immunity, specifically neutrophil recruitment [66]. In analyses of lipids related to AD, central position was given to FAs, with double bonds in the natural *cis* configuration. As oxidative stress is also documented in inflammation of AD, Ferreri et al. examined *cis-trans* isomerization of membrane unsaturated lipids catalyzed by free radicals in AD/eczema syndrome, and showed that *trans* FAs can arise from isomerization of natural *cis* lipids under conditions of radical stress—giving the *trans* FAs a role in perturbation of membrane properties, but also a role as markers of cellular stress [67]. 

As part of the ‘‘atopic march’’, AD is often accompanied by other atopic disorders, such as asthma, allergic rhinoconjunctivitis, food allergies, and eosinophilic esophagitis which may appear simultaneously with the onset of AD or later with its progression. Given that progression of atopic disease starts with skin inflammation, AD, management of atopic patients should not be concentrated just on the treatment of acute flares, but also be directed towards ameliorating epidermal barrier dysfunction and preventing active inflammation via maintenance therapy and preventive measures. 

### 7.1. Supplementation with ω-3PUFAs in Atopic Dermatitis

Given fish oil, which contains EPA and DHA, is reported to be useful for improving dermatitis symptoms, due to the ω-3 PUFAs overall suppressive, anti-inflammatory effects—inhibition of lymphocyte proliferation, cytokine and antibody production, adhesion molecules expression, NKCs activity and triggering apoptosis [68,69]. For instance, fish oil supplementation during pregnancy was associated with improvements in clinical severity in AD in the first year [70]. The early nutrition is crucial for the development of the infant’s immune system. Many studies have shown that breastfeeding has a protective effect on the development of AD [71,72]. An increased incidence of AD was found in infants consuming breast milk rich in saturated fat and low in ω-3 fats [73]. This fact emphasizes the importance of DHA and EPA in early infant development. Therefore, the US Food and Drug Administration included the LC-FAs, DHA, and ARA in infant formulas. The cutaneous dryness and pruritus were reduced by oral supplementation of fish oil in rats [74]. Dry skin is a consequence of the epidermal water loss, due to SCBP defect [75]. After 60 days of fish oil consumption, a 30% increase in cutaneous hydration was detected, and the itch-related scratching behavior was eliminated. The 90-day supplementation resulted in increased uptake of DHA, EPA, and DPA into the skin. The application of PPAR activators in AD divides opinion. However, topical PPAR-α therapy shows a positive outcome in AD treatment. Few described mechanisms result in the maturation of SCPB. The most prominent are higher epidermal FAs, cholesterol, sphingomyelin production, and β-glucocerebrosidase activity [76]. DHA, EPA, and other natural PPAR agonists (such as *cis*-9, *trans*-11 conjugated linoleic acid (CLA) and luteolin, rosmarinic acid, biochanin A found in red clover) may be used as the anti-inflammatory agents in AD treatment [77]. These nutrients are known as “reversible agonists” of PPAR-γ and are dependent upon higher concentrations to activate the receptor. For instance, CLA is endogenously produced by the probiotic bacteria in the colon from LA. Therefore, the therapeutic effect of probiotics in AD may be a result of CLA production [78]. In the recent decade, endogenous lipid mediators, resolvins, attract scientists’ attention. These mediators are generated during the acute inflammation resolution phase from n-3 PUFAs. Resolvin E1 (RvE1; 5S,12R,18R-trihydroxy-eicosapentaenoic acid) is a member of the E series of resolvins, which is isolated from EPA [79,80,81,82]. RvE1 significantly reduces DCs migration, interleukin IL-12 production, neutrophil transendothelial migration, and dermal inflammation in complex disease models [83,84,85]. In another animal model, NC/Nga mice, RvE1 reduced the development of AD-like skin lesions induced by 2,4-dinitrofluorobenzene (DNFB) treatment by suppressing the production of IL-4 and IFN-γ by activated CD4+T cells, and by decreasing lesional infiltration by CD+4, CD8+ T cells, as well as the mast cells and eosinophils, in addition to suppression of total serum IgE levels [86]. In the treatment of AD, the use of DHA and EPA may also ameliorate inflammation by modulation of TLR-2 and TLR-4 and subsequent signaling [53]. Recent research led by Nishi et al. showed that docosahexaenoyl ethanolamide (DHEA), an endogenously produced DHA metabolite, mitigates IgE-mediated allergic reactions in vitro and in vivo by inhibition of mast cell degranulation which indicated that DHEA could be a promising anti-allergic agent [87]. To summarize, ω-3 PUFAs, ALA and corresponding derivates, have numerous beneficial effects for the preventive or therapeutic use in AD patients, such as barrier function maintenance, stratum corneum maturation and differentiation, lamellar body formation, LOX and proinflammatory eicosanoid inhibition, cytokine suppression, inhibition of mast cell degranulation and modulation of other immune cells [68,87,88]. 

### 7.2. Supplementation with ω-6PUFAs in Atopic Dermatitis

As from Burr and Burr findings and further attempts to substitute deficient EFAs with relatively high daily oral doses of LA oils in patients with AD/eczema, ω-6 PUFAs supplementation, primarily GLA dietary supplementation and its capacity to relieve signs and symptoms of AD were investigated in numerous studies with contradictory outcomes [89,90]. Early studies showed that GLA could be a dietary supplement to ameliorate dry skin and AD by modifying FAs metabolism and improving SCPB, however, more recent meta-analyses and reviews raised doubts about earlier findings and the effectiveness of GLA-rich supplementation (mostly EPO, borage oil that contains three times more GLA than EPO) in the therapeutic management of AD [22,90,91,92]. Because of the skin’s inherent requirement for GLA and its inability to synthesize it, GLA supplementation via ingestion of GLA-rich oils or its topical administration are becoming more popular among healthy adults, as well as patients with impaired skin barrier, because of its beneficial effects on the biophysical skin parameters—skin moisture, TEWL, firmness, roughness, elasticity [91,93,94]. One clinical study involved 130 subjects with mild AD who were administered GLA-rich oil into a diet for oral consumption [95]. In a four week period, which is the minimum period for middle-aged epidermis to turn over and FAs to reach new tissue concentrations, the GLA group showed lower TEWL and a higher stratum corneum index compared to the control. The review of Foster et al. identified 12 clinical trials of topical or oral borage oil for treatment of AD and one preventive trial with highly variable results and the conclusion that supplementation with borage oil could be of benefit in some patients with less severe AD as additional treatment, but could not be used as monotherapy, due to the lack of greater clinical effect, especially in patients with more severe AD [90]. The possible generation of anti-inflammatory metabolites from GLA might be a reasonable explanation of the mechanism of skin barrier recovery. GLA enters the ω-6 biosynthesis pathway distal to the Δ6-FADS enzymatic step and is rapidly converted by FAs elongase 5 (ELOVL5) to measurable levels of DGLA, which can be further converted to metabolites with predominantly an anti-inflammatory role (PGE1 and 15-HETrE) [23]. Both PGE1 and 15-HETrE antagonize the synthesis of AR-derived pro-inflammatory eicosanoids, but only PGE1 is involved in T-cell maturation and differentiation; thus, IgE production may be influenced by PGE1, and alleviation of AD may be attained. One would raise the question of how does not GLA supplementation lead to a pro-inflammatory state, due to the increase in pro-inflammatory ARA, which is further synthesized from DGLA utilizing Δ5-FADS enzymatic activity. That is because of differential expression of enzymatic activities within organs, tissues and inflammatory cells (i.e., human neutrophils contain ELOVL5, but not FADS1)*,* but also population/ethnicity-based genetic and epigenetic variations within the *FADS* cluster which are responsible for the amount of tissue and circulating LC-PUFAs levels [22,96]. Skin also appears to have high ELOVL5 activity comparing to Δ5-FADS activity [97].

As GLA supplementation shows positive effects on the reduction of lipid pro-inflammatory mediators and amelioration of clinical symptoms of chronic inflammatory disorders, not only AD, there are promising strategies to maintain these anti-inflammatory effects without a consequent marked increase in ARA with its potentially harmful effects. These supplementation strategies are based on the addition of ω-3 LC-PUFAs, EPA, and DHA, to GLA-enriched supplement [98]. There are few rationales for combined ω-3 LC-PUFAs, and GLA enriched supplementation: (a) ω-3 LC-PUFAs inhibit the conversion of GLA metabolite DGLA to ARA; (b) this combination inhibits LT production, as well as the genes for pro-inflammatory cytokines; (c) addition of ω-3 LC-PUFAs enriches cells and tissues with EPA, DPA, and DHA and their potent anti-inflammatory metabolites [22]. Combinations of botanical oils rich in ω-6 GLA (like borage oil, EPO) and ω-3 PUFAs ALA and SDA (like linseed oil, flaxseed oil), also significantly increase levels of DGLA without increase on circulating ARA levels. This supports prior findings that not only conversion of GLA to DGLA is enhanced, but also further conversion of DGLA to ARA is inhibited when providing combined ω-3 and ω-6 supplementation. One of the most valuable oils is Echium oil extracted from the seeds of *Echium plantagineum* which contains high amount of GLA (19%), but also ALA (10%) and SDA (13%) which when consumed and being further metabolized increases plasma concentrations of EPA, DPA, and DGLA without increasing the ARA levels [99]. This oil is a good alternative for people who cannot tolerate fish oil or for vegetarians, to benefit from enhanced anti-inflammatory effects of ω-6 and ω-3 PUFAs. 

Despite the confusing pathophysiological pathway, lipid and other nutritional treatments may help the maturation of the SCPB and reduce the risk of AD. Nutritional modulation begins already during gestation. Low levels of ARA in cord blood have been associated with the risk of dermatitis [100,101,102]. Furthermore, ARA levels were in a positive correlation with gestational length. ARA is the precursor to pro-inflammatory eicosanoids, series-4 LT, and lipoxin. On the other hand, ARA is a precursor to PGE2, which is involved in the maturation of regulatory T cells, which are essential for inflammatory response reduction [103]. Trans-FAs, found in margarine and baked goods, inhibit the Δ5- and Δ6-FADS enzymes, which are crucial for the LA and ALA conversion to LC products. Concentrations of cis- and trans- PUFA isomers in cord blood are equated to maternal serum concentrations. However, there is an inverse relation between infant trans-FAs, ARA, and DHA concentrations in serum [104]. Trans-FA intake deprivation during pregnancy may help reduce the risk of AD by increasing the concentration of ARA in infant serum. Also, an extended gestational period likely gives the epidermis more time to mature. Infants can obtain adequate levels of ARA from infant formulas (*Mucor javanicus* is used as the source), as well as egg lecithin [12]. In a recent paper, Blaess et al. emphasized the fact that despite new targeted-oriented anti-inflammatory drugs, we still lack personalized therapy and prophylaxis that would be a valuable complementary treatment option to restore the skin, enhance the SCPB, and ameliorate dry skin and associated pruritus. They propose a new safe topical therapeutic combination of LA and amitriptyline to treat dry skin, as well as mild to moderate AD, which would also prevent the AD relapses [105]. 

## 8. Psoriasis and PUFAs

Psoriasis is a systemic inflammatory disease of multifactorial improve, in which an increased release of proinflammatory cytokines and chronic activation of the immune system cause damage to various tissues and organs [106]. It is a T-cell mediated disease affecting 2–3% of the population, with chronic inflammation of the skin, but not limited to it, caused by increased levels of lesional and circulating inflammatory Th17, Th22, and Th1 cells which elaborate IL-17, IL-23, IL-22, and IFN-γ cytokines, respectively [107]. There is high TLRs expression detected in tissues of psoriasis patients, as well as in peripheral blood mononuclear cells (PBMCs), which activation has been related to resistance to pathogenic microorganisms, but also exacerbation of the disease. This is demonstrated by the reaction seen after application of topical TLR-7/8 agonist, imiquimod, which is a potent immune activator that exhibits its pro-psoriatic actions via the well-known IL-23/IL-17 axis [108,109]. 

Psoriasis is characterized by erythematous, scaling lesions, with distinct clinical phenotypes: vulgar, inverse, guttate, erythrodermic, and pustular [110]. The gold standard in assessing psoriasis severity is the Psoriasis Area and Severity Index (PASI) score [111]. Although the etiology of psoriasis is still not completely understood, various risk factors have been recognized, including a strong genetic component and environmental risk factors, such as diet, obesity, medications, smoking, trauma, and stress [112]. It is estimated that 73% of psoriasis patients have at least one comorbidity. Studies have demonstrated the association of psoriasis with psoriatic arthritis, inflammatory bowel disease, cardiovascular diseases, depression, obesity, type 2 diabetes, and metabolic syndrome [113]. Systemic inflammatory state led by IL-23/IL-17 pathway, a central player in immune regulation, and the related Th17 cells seem to be the common denominator for all these comorbidities, as it is for many other chronic inflammatory diseases [114,115].

Psoriasis patients may have disrupted lipid and amino acid metabolism [116]. The disease is characterized by abnormal keratinocyte hyperproliferation, which arises due to the activation of T cells, subsequently leading to the production of ARA, which leads to the generation of various pro-inflammatory mediators. These include PG, LT, cytokines, and adhesion molecules [117]. In comparison to healthy skin, psoriatic plaques show higher levels of ARA and its metabolites—LTB4 and 12-hydroxyeicosatetraenoic acid (HETE), which are chemotactic for neutrophils [118]. Significant advancements in the understanding of psoriasis immunopathogenesis in recent years led to the development of various therapeutic options for this disease. Topical therapy does not affect systemic inflammation present in psoriasis, and all the currently available treatments have to be administered continuously, which increases the risk of side-effects. Therefore, new treatment modalities of high efficacy, low probability of adverse events, and low cost are necessary. Only a few studies are exploring the influence of lifestyle factors on psoriasis pathogenesis, and no guidelines regarding dietary recommendations. Evidence is accumulating that by lifestyle modifications and incorporating food components, such as EFAs it is possible to produce standalone benefits among sufferers and improve psoriasis severity [119]. 

Psoriasis is not common in Africans, probably partly, due to genetic factors. However, the dietary habits may provide another explanation, since diet in most parts of Africa is high in LA, but low in other PUFAs and riboflavin [120]. In psoriasis patients, ALA derivatives, DHA and EPA, can modulate the epidermal immune response by influencing T lymphocytes, acting on TLRs (TLR-2 and -4), and activating caspase cascades that have an impact on various inflammatory dermatoses, such as prior mentioned AD [53]. LA, the precursor of PGE2, and its high intake, especially in the absence of other PUFAs and riboflavin, results in high tissue production of PGE2, which is known to suppress cellular immunity, resulting in decreased expression of psoriasis [120]. Similarly, a low prevalence of psoriasis in Eskimos has been attributed to the high dietary intake of EPA from fish and marine mammals. However, even when on a Western diet, Eskimos have plasma ARA levels far below those seen in Europeans, while DGLA levels are higher in Eskimos. That seems to be due to a genetic abnormality in FAs desaturation, since they are found even when EPA intakes are low [121]. 

Partly due to the hypothesis that overproduction of pro-inflammatory eicosanoids from ARA are central to the pathogenesis of psoriasis, and partly due to the observation that Eskimos have a low incidence of the disease, a number of studies have examined the effect of dietary supplementation with fish oils [55], since fish oil is rich in LC ω-3 PUFAs, particularly EPA and DHA [122]. Ω-3 PUFAs exert multiple functions in humans and are crucially involved in limiting and resolving inflammatory processes. They have been intensively studied for their ability to improve morbidity and mortality in patients with cardiovascular disease [123], possibly from reductions in platelet aggregation, blood viscosity, thrombotic tendency, serum triglycerides, and cholesterol [124]. Due to their anti-inflammatory and anti-chemotactic properties, PUFAs have been used as a safe, adjunct therapy in various skin diseases [125]. Inhibition of inflammation by ALA and the derivatives are based on barrier function maintenance, stratum corneum maturation and differentiation, proinflammatory eicosanoid inhibition, lamellar body formation, lipoxygenase inhibition, and cytokine suppression [126]. ω-3 PUFAs attenuate cutaneous inflammation by competing with the inflammatory ARA and inhibiting pro-inflammatory eicosanoid production [88]. The mechanism of action of fish oil supplementation in psoriasis treatment is based on the alteration of serum, epidermal- and blood cell-membrane lipid composition [127]. Exogenous supplements of ω-3 PUFAs are incorporated into cell membranes where they compete with ω-6 PUFAs as substrates for the same 5-, 12-, and 15-LOX and COX pathways [124]. The metabolites of ω-3 PUFAs are far less potent inflammatory mediators than the degradation products of ARA [127], and they are involved in the feedback regulation of IL-1 [124]. ω-3 PUFAs suppress the production of pro-inflammatory cytokines, such as IL-6 and TNF-α [128], which are known to be increased in psoriasis patients [129].

Using a fat-1 transgenic mouse model, Quin et al. investigated the mechanisms involved in preventing inflammation in psoriasis-like mice by ω-3 PUFAs. The results showed that ω-3 FAs acted on Th17 cells to produce lower levels of inflammatory factors, including IL-17, IL-22, IL-23, and stimulated Treg cells to produce higher anti-inflammatory factors, such as Foxp3 [108]. A study from 2018 [130], which investigated the effects of a ω-3 derived metabolite resolvin E1 (RvE1) on psoriatic dermatitis, using an imiquimod-induced mouse psoriasis model, showed that RvE1 potently suppressed the inflammatory cell infiltration and epidermal hyperplasia in the psoriatic skin. RvE1 has shown to inhibit IL-23 production by DCs in vitro, as well as the migration of cutaneous DCs and IL-17-producing cells. These suppressive, antipsoriatic effects of RvE1 are as well mediated through inhibition of LTB4 and its receptor BLT1, which is also known to be expressed on neutrophils, the cells that are increased in psoriatic skin lesions. Resolvins regulate neutrophil migration in both animals and humans so that could be one of the main anti-inflammatory effects of ω-3 FAs on psoriasis [130]. Although animal studies have supported the therapeutic effect of ω-3 PUFAs on psoriasis-like lesions, investigations on humans have shown contradictory results [53]. Studies show that adding fish oil to the diet of psoriasis patients leads to an increase in the plasma and platelet EPA-to-ARA ratios and, by in vitro studies, to a significant decrease in LTB4 synthesis by neutrophils [131]. Also, dietary intake of very LC ω-3 FAsmay suppress the expression of CD25 positive lymphocytes, which may partly account for their anti-inflammatory effect [132]. Depending on the dose, supplementation with ω-3 FAs results in inhibition of various pro-inflammatory mediators [117]. In 2017, the first systematic review of the effects of ω-3 PUFA intervention on psoriasis severity was published by Upala et al. [125]. Based on the data of 12 relevant studies, the results of this systematic review were inconclusive regarding improvements in PASI score, erythema, scaling, itching, and infiltration. Due to the potential to improve psoriasis, but controversial earlier findings, Clark et al. [117] have recently conducted a meta-analysis to evaluate the efficacy of ω-3 FAsas a monotherapy in treating psoriatic patients. The results showed that ω-3 PUFA supplementation, as a monotherapy, elicited significant reductions in PASI score, erythema, and scaling. In a subgroup analysis, higher dosages of >1800 mg/day and <8 weeks in duration were associated with more beneficial outcomes. On the other hand, supplementation with ω-3 PUFA did not improve itching, desquamation, and infiltration. In patients treated with ultraviolet B (UVB) phototherapy, ω-3 FAs prolonged the beneficial results of the therapy [133].

Additional benefits of ω-3 PUFAs supplementation in patients with psoriasis are their possible hypolipidemic effects and prevention of the development of obesity and insulin resistance [134]. Since psoriasis patients have a higher prevalence and incidence of obesity than the general population [135], reduction of the inflammatory burden associated with an increased amount of fat tissue could certainly contribute to psoriasis improvement. That is based on the fact that rapamycin complex 1 (mTORC1) signaling, crucial for the regulation of T-cell homeostasis, but also proinflammatory signaling of keratinocytes through activation of NF-κB pathway, is activated via increased systemic circulating obesity-related inflammatory cytokines like TNF-α, IL-6, and adiponectin released from visceral fat [136]. Fish oil supplementation may also improve possible side effects of cyclosporin therapy—hypertension and nephrotoxicity, and lower serum triglycerides and cholesterol, which could be especially helpful in patients taking long-term retinoid therapy [124].

EPO has shown beneficial effects in psoriasis. It contains a very high percentage of LA (70–74%) and GLA (8–10%), which are precursors of anti-inflammatory eicosanoids, such as the series 1 PGs and 15-HETrE. 15-HETrE blocks the conversion of ARA to LTB4 by direct inhibition of 5-LOX, while GLA suppresses inflammatory cytokines—IL-1β, IL-6, and TNF-α [137], which mediate systemic inflammation and are increased in serum of psoriatic patients [138]. 

PPARs have an important role in psoriatic patients, since they exhibit antiproliferative and immunomodulatory functions in the skin [139]. In human keratinocytes, expression of PPAR subtypes α and γ is reduced, while the expression of β subtypes is increased [140]. In animal models, activators of these receptors, such as OA and LA, accelerate the development of skin barrier and maturity of stratum corneum [141]. These findings were supported in humans by an improvement of psoriatic plaques in patients treated with troglitazone [140]. When pioglitazone—a PPAR-γ agonist was evaluated in patients with psoriatic arthritis, improvement of swollen joints and a 38% reduction of PASI was observed, as well as adverse events, such as limb edema and weight gain [142]. Although systemic administration has shown success, the topical application of PPAR agonists was not effective in psoriatic patients [53]. Therefore, to be beneficial, the activation of PPARs must be systemic and not local [139]. It seems that in psoriasis treatment, high-dose EFA therapy may be a safer alternative to PPAR-γ agonists [53]. 

With regard to topical FAs application, a topical linoleic acid-ceramide moisturizer was evaluated in a randomized control trial, and results show that it can alleviate psoriasis, and could be useful for the treatment and prevention of psoriasis [143]. A study aimed to investigate the in vitro and in vivo efficacy of petroleum ether extract of *Annona squamosa* seeds (ASO) as an antipsoriatic agent was conducted recently [144]. The extract predominantly consists of PUFAs (LA and OA) and shows higher inhibition of keratinocyte proliferation compared to corticosteroid clobetasol propionate (CP) in mice. After the application of ASO, there was a decrease of lesional inflammatory cytokines (IL6, IL17, TNF-α, INF-γ, GM-CSF), and no adverse events were noted, which warrants further investigations of this novel topical antipsoriatic agent for therapy in humans.

PUFA supplementation may lead to improved psoriasis management and the prevention of comorbidities. Even a mild improvement in psoriasis severity mediated by PUFA could decrease the dosage of currently available therapies and reduce the risk of their side effects. Therefore, further clinical research and development of new treatment options and guidelines based mostly on prior mentioned beneficial supplementation with both ω-3 and ‘’good’’ ω-6 PUFAs are needed to optimize the therapeutic approach in patients with psoriasis.

## 9. Acne and PUFAs

Acne vulgaris is a chronic inflammatory skin disease affecting pilosebaceous follicles. It has been estimated that approximately 85% of adolescents have acne; however, this dermatosis may occur in childhood and can persist beyond adolescence. Clinically, it is characterized by open and closed comedones and inflammatory lesions (papules, pustules, or cysts), located on the skin areas rich in sebaceous glands, including the face, chest, and back [145]. Acne is related to significant psychological morbidity, which may occur not only at the acute phase of the disease, but also in adulthood because of permanent scarring. Therefore, adequate and early treatment is necessary to prevent scarring and psychological sequelae [146]. 

Pathophysiology of acne involves the interplay between four main pathogenic factors, including overproduction of sebum, altered follicular keratinization, follicular colonization with *Cutibacterium acnes* (formerly *Propionibacterium acnes*) and inflammation that engages both innate and acquired immunity [147]. Moreover, several additional factors are implicated in the pathogenesis of acne, including genetic factors, neuroendocrine mechanisms, and diet [148,149]. 

An association between diet and acne has been hypothesized for decades. The epidemiological studies have shown that the incidence of acne is significantly lower in non-industrialized societies than in westernized populations [150]. For example, acne is extremely rare among the Inuit, Okinawan, and Kitavian Islanders, and Ache hunter-gatherers. Moreover, several studies have noted that, as these populations made their transition to modern life, either through a local cultural change or relocation, adopting a Western diet, the prevalence of acne increased to similar ranges as in Western societies [151].

Typically, the western diet consists of a low ratio of ω-3 to ω-6 FAs, as compared with non-westernized food. In standard western diet, the ω-6 content is almost ten times greater than ω-3 content, mostly because of the low intake of fish, wild plants, and wild game. Additionally, ω-3 fats are usually lost or oxidized during food processing and cooking. Jung JJ et al. investigated the influence of dietary patterns on acne in Koreans. They found that patients with acne consumed less fish and more junk food when compared to healthy individuals [152]. Similarly, the study of an Italian population demonstrated that fish consumption had a protective effect in means of moderate and severe acne development [153]. The additional food types that promote acne are milk and dairy products and high glycaemic index carbohydrates [154].

The anti-inflammatory activity of ω-3 FAs have been recognized for decades, and their therapeutic effect in acne vulgaris occurs because the inflammation is one of the most important pathogenetic factors in acne. Since there was never any doubt concerning the role of inflammation in the late stages of acne, consistent with the occurrence of inflammatory lesions, later studies have shown that the inflammatory process is one of the earliest events in acne pathogenesis [154]. The latter has been confirmed by both increased infiltration of the perifollicular and papillary dermis by CD3+ and CD4+ T cells in uninvolved skin in acne patients and the effectiveness of the anti-inflammatory agents, such as topical benzoyl peroxide, dapsone, and antibiotics, in reducing non-inflammatory lesions (i.e., comedones) in acne patients [155]. 

ω-3 FAs, particularly DHA, inhibit dimerization of TLR-1 and TLR-2 signaling [156]. *C. acnes* increases the expression of TLRs on both keratinocytes and macrophages, which leads to hyperproliferation of keratinocytes and the initiation of inflammatory reaction. The activation of keratinocyte TLR-2 and TLR-4, induced by *C. acnes*, leads to activation of NF-κB and MAPK pathways, subsequently, IL-1, IL-6, IL-8, TNF-α, human β-defensin-2, granulocyte-macrophage colony-stimulating factor (GM-CSF), and matrix metalloproteinase (MMP) production [157,158,159]. Human β-defensin-2 belongs to the family of antimicrobial peptides (i.e., β-defensins), that are involved in the development of inflammation in acne. The β-defensins induce the release of proinflammatory cytokines and chemotaxis of immunocompetent cells and modulate cell maturation and migration [155]. *C. acnes*-induced activation of monocyte TLR-2 leads to the production of proinflammatory cytokines, including IL-1β, TNF-α, IL-8, and IL-12 [160]. Thus, DHA and EPA, inhibiting activation of TLR-signaling pathways, may decrease the inflammatory response in patients with acne.

Furthermore, ω-3 FAs inhibit the activation of the NLRP3-inflammasome in antigen-presenting cells. The activation of the NLRP3-inflammasome is triggered by *C. acnes*, which leaks out of the pilosebaceous follicle after the disruption of the follicular epithelium and gets into the contact with dermal macrophages. Subsequently, caspase-1 is activated, which leads to the release of monocytes-derived IL-1β [161]. IL-1β promotes Th17 activation, which leads to IL-17-mediated inflammation and keratinization. This process is regulated by reactive oxygen species (ROS) and proteases [157]. Snodgrass RG et al. have demonstrated that DHA inhibits the expression of pro-IL-1β and secretion of mature IL-1β in monocytes [156].

Additionally, it has been shown that EPA inhibits NF-κB activation. NF-κB transcribes proinflammatory cytokines, including IL-1, IL-6, IL-8, upon its activation by the interaction of *C. acnes* and TLR-2. EPA can also inhibit the expression of MMP-9, an endopeptidase that can degrade the components of the ECM and expand the inflammation [162].

Insulin-like growth factor-1 (IGF-1) signaling is one of the most critical pathways in acne development, which is sufficient to induce pro-inflammatory cytokine (IL-1β, IL-6, IL-8, TNF-α) and MMPs expression in human sebocytes [163]. Several studies have shown that ω-3 FAs decrease serum levels of IGF-1 and increase insulin-like growth factor binding protein-3 (IGFBP-3), thereby preventing IGF-1 from binding to its receptors on sebocytes and acroinfundibular keratinocytes [164]. Diets rich in refined carbohydrates, saturated fat, trans-FAs, milk, and dairy products enhance IGF-1/phosphoinositide 3-kinase (PI3K)/Akt/mTORC1 signaling [147]. IGF-1 signaling leads to the inhibition of FOXO1 (forkhead box class O1) and the activation of mTORC1 signaling. The inhibition of FOXO1 signaling leads to the de-repression of all significant transcription factors of sebaceous lipogenesis, including androgen receptor (AR), PPAR-γ, liver X receptor-α (LXR-α), and SREBP-1c. Significantly, ω-3 FAs inhibit SREBP-1c, which influences acne pathogenesis on several levels: (1) Stimulates the synthesis of sebum triglycerides which promotes the growth of *C. acnes*; (2) stimulates the synthesis of palmitic acid which activates TLR-2; (3) stimulates the activity of Δ6-FADS that is, together with Δ5-FADS, one of the key enzymes for the synthesis of unsaturated FAs, ARA and sapienic acid; (4) stimulates stearoyl-CoA desaturase which catalyzes the conversion of stearic acid to OA, subsequently inducing disturbed keratinocyte Ca^2+^ gradient which leads to the release of IL-1α, a cytokine that promotes comedogenesis [165]. We have mentioned sapienic acid, a product of Δ6-FADS on palmitic acid which has long been attributed to the sebaceous gland. It is important to point out that nowadays, sapienic acid is becoming a new marker for the FAs metabolism [166,167]. 

ω-3 FAs compete with ARA for embedding into the PL of the cell membrane and acts as a substrate for the COX-2 and 5-LOX, which leads to the reduction of pro-inflammatory PGE2 and LTB4 [165]. Moreover, ω-3 FAs inhibit mTORC1 signaling, which subsequently downregulates SREBP-1c pathways [168]. 

**Figure 2:**
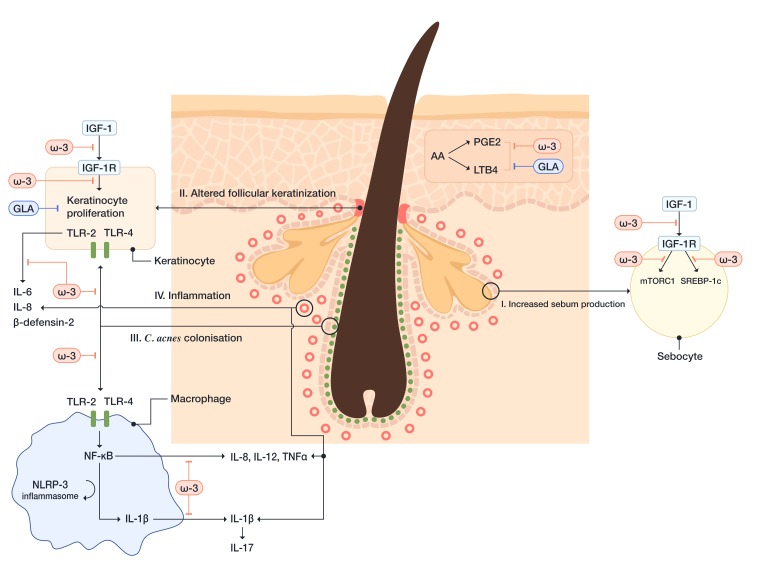
Schematic presentation of the mechanisms via which essential fatty acid influence the four main pathogenetic factors in acne, including I. increased sebum production, II. altered follicular keratinization, III. *Cutibacterium acnes* colonization, and IV. inflammation. ARA, arachidonic acid; *C. acnes*, *Cutibacterium acnes*; GLA, gamma-linoleic acid; IGF-1, insulin-like growth factor 1; IGF-1R, insulin-like growth factor 1 receptor; IL, interleukin; LTR4, leukotriene B4; mTORC1, mechanistic target of rapamycin complex 1; NF-κB, nuclear factor kappa B; PGE2, prostaglandin E2, SREBP-1c, sterol response element-binding protein-1c; TLR, Toll-like receptor; TNF-α, tumor necrosis factor-alpha; ω-3, omega-3 fatty acids.

Although ω-6 FAs are generally considered to have pro-inflammatory effects, GLA may improve acne not only because of its keratolytic properties, but also because of its anti-inflammatory activity. The GLA is converted to DGLA, which can reduce follicular hyperkeratinization via 15-HETrE, by modulating nuclear MAPK/activator protein 1 (AP-1)/apoptotic signaling pathways [169]. Besides, DGLA inhibits 5-LOX, the crucial enzyme for the conversion of ARA to proinflammatory eicosanoids, such as LTs C4 and B4 [22] (Figure 2).

Based on the abovementioned findings and the ones discussed in the AD section, it can be expected that foods rich in ω-3 PUFAs and GLA may improve acne. However, a few human studies have been conducted to evaluate the effect of dietary supplementation with these PUFAs on acne. 

An epidemiological study conducted in 1961 in North Carolina among more than 1000 adolescents, found that the participants who consumed large amounts of fish and seafood, both rich in ω-3 FAs, had less oily skin and fewer acne lesions, including blackheads, papules, and pustules. Rubin et al. conducted a small study of five patients with mild to moderate acne vulgaris, who consumed dietary supplementation, consisted of 250 mg of EPA obtained from sardines and anchovies, 3.75 mg of zinc gluconate, 50 mcg of selenium, 50 mcg of chromium and 50 mg of EGCG from green tea extract. The dosage was four capsules per day. After two months, four of five patients experienced a decrease in both total lesions count, and inflammatory lesions count. The average total lesion count decreased from 62.8 to 40.4, and the average inflammatory lesion count dropped from 20.8 to 6.8 [170].

Moreover, Khayef et al. examined 13 males with inflammatory acne who consumed three capsules of fish oil daily for 12 weeks, which contained a total of 930 mg of EPA, 720 mg DHA, and 174 mg DPA. Their other dietary habits and existing acne treatment remained unchanged. The results demonstrated that acne improved in eight patients, remained unchanged in one, and worsened in four patients. The authors raised the question of whether the efficacy of fish oil supplementation is dependent on acne severity, since 7/8 patients who experienced the improvement of acne initially had moderate or severe acne [171]. Jung JY at al. performed a randomized, double-blind prospective study that included 45 patients with mild to moderate acne. The patients were assigned to the three groups, including ω-3 FAs group (taking 2000 mg of EPA and DHA daily), a GLA group (taking borage oil containing 400 mg GLA daily), and a control group (not given or received any treatment). After ten weeks of intervention, both ω-3 FAs group and a GLA group showed a significant decrease in the mean inflammatory and non-inflammatory acne lesion count. There was no significant difference between those two groups regarding mean inflammatory or non-inflammatory acne lesion count. The histologic evaluation demonstrated a reduction in inflammation in all treatment groups. Immunohistochemical analysis revealed a decreased expression of IL-8 in both ω-3 FAs group and a GLA group. Apart from its pro-inflammatory properties, IL-8 promotes follicular hyperkeratinization and epidermal hyperplasia. The researchers concluded that moderate doses of ω-3 FAs or GLA could improve acne lesions. Costa et al. conducted a study to evaluate the influence of systemic antibiotic treatment and oral supplementation of FAs on FAs levels in the sebum of patients with acne. The study comprised 45 male patients with acne who were divided into three treatment groups. The first group was treated with lymecycline (300 mg per day); the second group received 540 mg of GLA, 1200 mg of LA, and 510 mg of OA per day; and the third group received the combination of the treatments used in the first and the second group. All therapeutic regimens were taken for 90 days. The results have shown that there were slight changes in patients’ sebum composition in all groups. Still, neither lymecycline nor FAs supplementation was able to markedly influence the fatty acid contents of the sebum in patients with acne [168]. 

Protective effects of GLA on skin barrier and maintenance of its hydration are demonstrated in acne patients who were treated with isotretinoin, the medication that is the first-line therapy in the treatment of severe and refractory acne. Due to its sebo-suppressive activity, isotretinoin treatment is associated with skin dryness and cheilitis in practically all patients. Park KY et al. evaluated the effect of EPO supplementation containing GLA on xerotic cheilitis in acne patients was with isotretinoin. The study included 40 patients with acne treated with isotretinoin at daily doses between 0.25 and 0.4 mg/kg for eight weeks. Half of the participants simultaneously received six 450 mg EPO capsules trice daily. The results demonstrated lower TEWL and higher corneometry values of the lips in the group receiving EPO in addition to isotretinoin [172]. 

Moreover, isotretinoin treatment is often associated with an increase in serum triglyceride levels. Khrishna et al. demonstrated that ω-3 FAs supplementation stabilized the expected rise in triglyceride levels in patients who were treated with isotretinoin and have had pre-existing hypertriglyceridemia [173]. 

## 10. Conclusions

While major changes have taken place in the human diet over the past 10,000 years, our genes have not changed that much. As humans are naturally used to food on which they evolved, and their genetic patterns were established—therefore, it is not surprising that newly established Western diets deficient in ω-3, and rich in ω-6 PUFAs promote the pathogenesis of many chronic inflammatory (skin) diseases. ω-3 PUFAs, DHA, and EPA, are associated with healthy aging and have a role in the prevention and treatment of numerous diseases by exerting their beneficial effects through inhibiting actions of multiple cytokines in disease progression. On the other side, ω-6 FAs, ARA with its eicosanoids, have opposing properties from those of ω-3 PUFAs and change the physiological state to pro-inflammatory with increased production of inflammatory LTs, PGs, and cytokines. As things are usually more gray than just black or white, we could not simply divide ω-3 and ω-6 FAs to ‘’good’’ and ‘’bad’’ omegas because there is a lot of scientific and clinical evidence of benefits of GLA supplementation in the treatment and prevention of chronic inflammatory diseases. Although there has been a pile of articles related to the application of ω-3 and ω-6 PUFAs in most common skin diseases, their results and conclusions were mostly conflicting and ambiguous, which could be explained by the fact that different studies examined diverse sources, doses, and duration of omega supplementation. There are more and more promising studies in favor of combined GLA and ω-3 LC-PUFAs supplementation that shows the highest potential in diminishing inflammatory processes and ameliorating chronic inflammatory diseases, as well as the chronic inflammatory skin diseases like AD, psoriasis, acne, and to some extent hidradenitis suppurativa. Based on mounting evidence that revealed significant population and individually-based genetic and epigenetic variations that impact PUFAs, their metabolism, associated metabolites, gene expression, and subsequent pro- or anti-inflammatory responses that are differently driving the risk of disease in individuals exposed to various stressors, at the moment, we could not recommend strict dietary and supplementation strategies that will be appropriate for all. However, we could use lipidomics to help us establish the best PUFAs supplementation strategies. Well-balanced nutritional ω-3/ω-6 FAs ratio, mostly Mediterranean diet, which is proven to promote health and prevent disease formation, should be fundamental in the prevention and additional treatment of various skin diseases. Additional anti-inflammatory PUFA-based supplementation (DHA-, EPA-, GLA-containing oils or supplements) should be encouraged in a targeted manner for individuals in need to provide better management of skin diseases, better treatment outcomes in addition to standardized skin-targeted therapy, to decrease the dosage of currently available therapies and to reduce the risk of their side effects, but most importantly, to maintain and improve overall skin health. Therefore, further clinical research and development of new treatment options using both ω-3 and ω-6 PUFAs are needed to optimize the therapeutic approach in patients with inflammatory skin diseases. 

## Figures and Tables

**Table 1:** Contents of ω-3 and ω-6 fatty acids in selected plant and animal-based foods.

	Food Source	ω-3			ω-6			Reference
		ALA *	EPA *	DHA *	LA *	ARA *	DPA *	
Oil	corn	0.6	-	-	49.83	-	-	[13]
	sunflower	0.33	-	-	49.89	-	-	
	soybean	7.6	-	-	51.36	-	-	
	wheat germ	5.3	-	-	55.1	-	-	
	Canola	9.15	-	-	18.65	-	-	[14]
	Safflower	0.1	-	-	12.72	-	-	
	Flaxseed	53.37	-	-	14.33	-	-	
Fish oil	Salmon	-	13.3	18.23	-	-	2.99	[14]
	Sardine	-	10.15	10.66	-	-	1.97	
	Herring	-	6.28	4.21	-	-	0.62	
	menhaden	-	13.18	8.56	-	-	4.92	
Vegetables	lettuce, raw	0.15	-	-	0.06	-	-	[13]
	green broccoli, raw	0.11	-	-	0.03	-	-	
	brussels sprouts, raw	0.17	-	-	0.08	-	-	
Fish	salmon, raw	0.09	0.89	1.19	0.15	0.05	-	[13]
	herring, raw	0.19	1.09	1.01	0.22	0.1	-	
	sardine, raw	-	0.51	1.16	0.06	0.04	-	
	trout, raw	0.1	0.15	0.5	0.37	0.05	-	
	cod, dried	-	0.02	0.62	0.03	0.12	-	
Meat	lamb, lean meat	0.11	-	-	0.11	-	-	[13]
	pork, fat and lean meat, without visible fat	-	-	-	1.63	0.03	-	
	beef, veal, 4 months, lean meat, without visible fat	0.08	-	-	0.13	-	-	
Seeds	chia, dried	17.83	-	-	5.84	-	-	[14]
	walnuts, dried	6.64	-	-	34.02	-	-	[13]
	hazelnuts, dried	0.11	-	-	5.09	-	-	
	almond, dried	0.3	-	-	10.54	-	-	

* g/100g; ALA, α-linolenic acid; ARA, arachidonic acid; DHA, docosahexaenoic acid; DPA, docosapentaenoic acid; EPA, eicosapentaenoic acid; LA, linoleic acid; ω-3, omega-3 fatty acids; ω-6, omega-6 fatty acids.
